# Modified Widman Flap Surgery with Osseous Recontouring for the Management of Residual Periodontal Pockets in Stage II Periodontitis: A Three-Month Clinical Case Report

**DOI:** 10.7759/cureus.112437

**Published:** 2026-07-11

**Authors:** Mehvish Khan, Aman Narang, Roopse Singh, Mayur Kaushik

**Affiliations:** 1 Department of Periodontology & Implantology, Subharti Dental College and Hospital, Swami Vivekanand Subharti University, Meerut, IND

**Keywords:** clinical attachment gain, modified widman flap, osseous recontouring, periodontal surgery, periodontitis, residual pocket

## Abstract

Periodontal pockets persisting after non-surgical periodontal therapy may require surgical intervention to facilitate adequate access for root debridement and elimination of inflamed pocket tissues. Modified Widman flap surgery remains a predictable periodontal surgical procedure that allows thorough debridement while preserving soft tissue architecture. A 45-year-old female patient presented with pain in the gums during mastication in the maxillary right quadrant. Clinical examination revealed residual periodontal pockets measuring 5 mm extending from the maxillary right central incisor to the first molar following completion of Phase I periodontal therapy. Bleeding on probing was present, and the clinical attachment level measured 4 mm. The patient was systemically healthy and had no history of smoking. A diagnosis of Stage II periodontitis was established. Modified Widman flap surgery with osseous recontouring was performed under local anesthesia. Following internal bevel and crevicular incisions, a full-thickness mucoperiosteal flap was reflected. Inflamed pocket epithelium and granulation tissue were removed, root surfaces were thoroughly debrided, and osseous recontouring was performed to establish physiologic architecture. The flap was stabilized using 3-0 polyglycolic acid/polylactic acid (PGA/PLA) sutures. Healing was uneventful throughout the postoperative period. At three months, probing pocket depth was reduced, and gain in clinical attachment level was observed; also, bleeding on probing was absent. Minimal postoperative gingival recession, mobility, or furcation involvement was observed. Modified Widman flap surgery combined with osseous recontouring provided favorable clinical outcomes in the management of residual periodontal pockets, resulting in reduced probing depths, attachment gain, and maintenance of soft tissue architecture after three months.

## Introduction

Periodontitis is a chronic multifactorial inflammatory disease associated with dysbiosis and characterized by progressive destruction of the tooth-supporting structures, including the periodontal ligament and alveolar bone [1]. The primary objective of periodontal therapy is to arrest disease progression, eliminate inflammation, and preserve the natural function and structures of the dentition. Non-surgical periodontal therapy consisting of scaling and root planing remains the major part of periodontal treatment and is effective in reducing inflammation and improving periodontal health [2]. However, deep periodontal pockets may persist even after proper instrumentation, and residual pockets greater than 4 mm are considered risk factors for disease recurrence and may require surgical intervention [3].

Periodontal flap procedures provide direct visualization of root surfaces and osseous defects, thereby helping in the complete removal of subgingival deposits and diseased soft tissues [4]. Among the various periodontal flap procedures, the modified Widman flap, originally described by Ramfjord and Nissle, remains a well-established and widely utilized access flap procedure for the management of persistent periodontal pockets requiring surgical debridement [5].

The biological rationale of the modified Widman flap lies in the removal of pocket epithelium and inflamed connective tissue, followed by close adaptation of the flap to the root surface, hence creating favorable conditions for periodontal healing [6]. Several studies have shown significant reductions in probing pocket depth and gains in clinical attachment following modified Widman flap surgery [7].

Irregular osseous contours are commonly associated with periodontal destruction and may contribute to already existing periodontal pockets. Osseous recontouring, when indicated, helps establish physiologic architecture, which helps in improving soft tissue adaptation and facilitates better oral hygiene maintenance [8]. The combination of Modified Widman flap surgery and osseous recontouring remains a valuable treatment modality for managing residual periodontal pockets following non-surgical therapy [9].

The present case report describes the successful management of residual periodontal pockets in a patient with Stage II grade B periodontitis using modified Widman flap surgery combined with osseous recontouring and evaluates the clinical outcome after three months.

## Case presentation

A 45-year-old female patient reported to the Department of Periodontology with a chief complaint of pain in the gums while eating in the upper right posterior region. The patient's medical history was non-contributory, and there was no history of tobacco use or smoking. A comprehensive periodontal examination was performed following completion of Phase I therapy, which included oral hygiene instructions, scaling, and root planing over multiple visits. Despite completion of Phase 1 therapy, periodontal pockets persisted in the maxillary right quadrant involving teeth 11 to 16. Although the gingival tissues appeared relatively well maintained clinically following completion of Phase I therapy, residual probing pocket depths of 5 mm with bleeding on probing persisted at the affected sites, indicating unresolved subgingival inflammation. The clinical attachment level was measured at 4 mm from the cemento-enamel junction to the base of the pocket. No tooth mobility or furcation involvement was detected. Based on clinical findings, generalized Stage II Grade B periodontitis according to the 2017 World Workshop Classification is based on interdental clinical attachment loss of 3-4 mm, maximum probing depth of 5 mm, radiographic bone loss confined to the coronal one-third (< 33%), and absence of tooth loss due to periodontitis [1].

Considering the persistence of periodontal pockets following non-surgical therapy, surgical treatment options for persistent periodontal pockets include open flap debridement, modified Widman flap, apically positioned flap with or without osseous resection, and regenerative procedures depending on defect morphology and treatment objectives. In the present case, a conservative modified Widman flap combined with localized osseous recontouring was selected because the primary objective was to establish physiologic osseous architecture while preserving the existing gingival contour.

After obtaining informed consent, the surgical site was anesthetized using a local anesthetic solution containing adrenaline in standard concentration.

**Figure 1:**
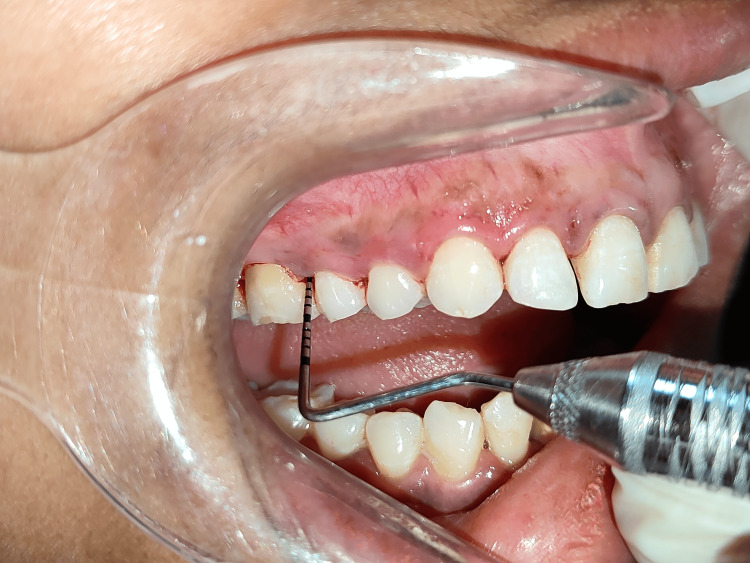
Preoperative pocket probing depth was measured using a University of North Carolina-15 (UNC-15) periodontal probe, showing a pocket depth of 5 mm.

**Figure 2:**
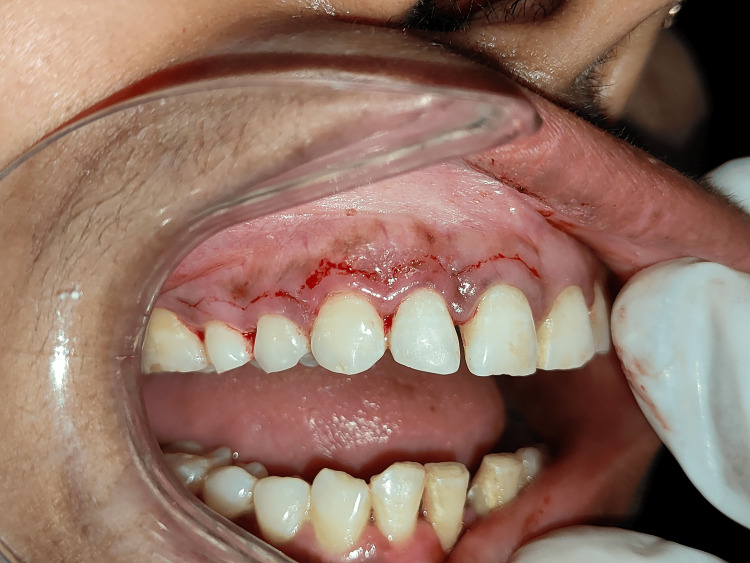
An internal bevel incision was placed close to the gingival margin, following the contour of the gingival tissues to facilitate excision of the pocket lining while preserving healthy gingival tissue extending from the mesial papilla of the central incisor to the mesial papilla of the first molar.

**Figure 3:**
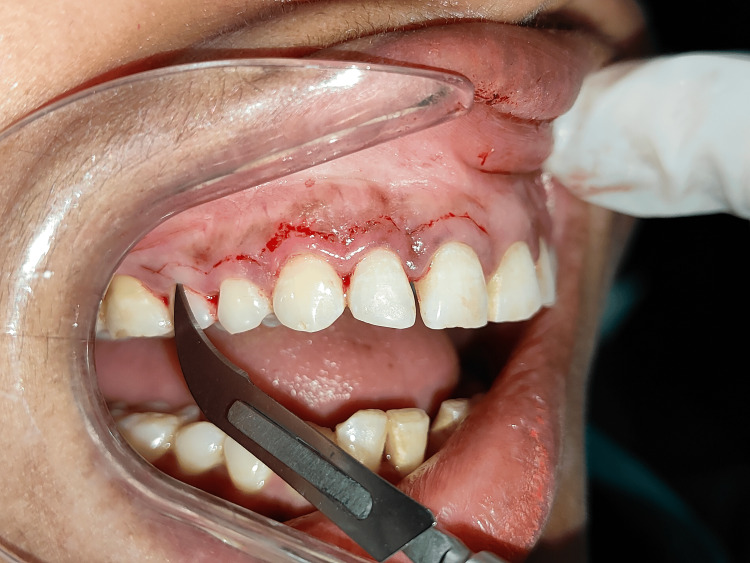
Following the internal bevel incision, a crevicular incision was made using a No. 12 blade from the mesial papilla of the central incisor to the mesial papilla of the first molar.

**Figure 4:**
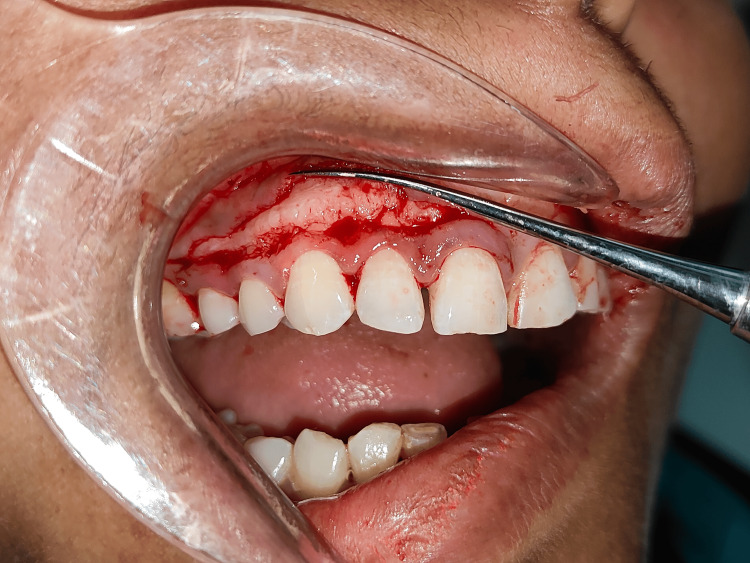
A full-thickness mucoperiosteal flap was reflected using a Glickman No. 6 periosteal elevator (P24G; Hu-Friedy, Chicago, IL, USA) following the incisions, exposing the underlying bone in the incised area.

**Figure 5:**
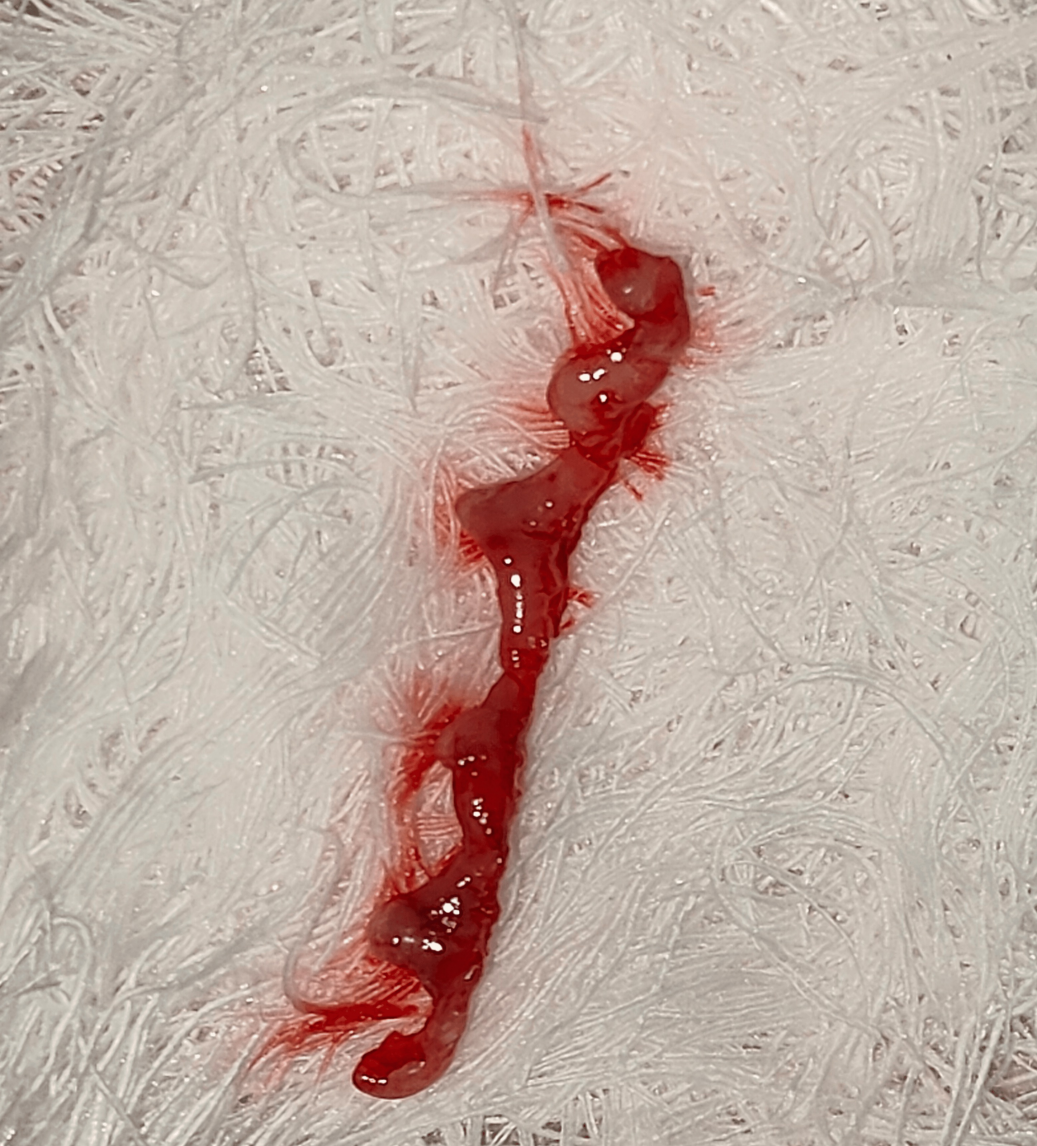
The inflamed pocket lining was removed following the flap reflection.

**Figure 6:**
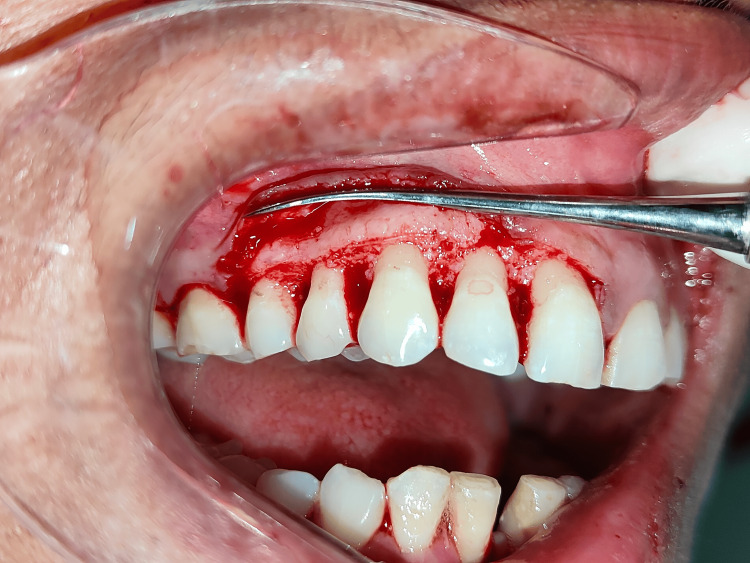
After removal of the inflamed lining, the hard tissue surface was curetted for the removal of any residual granulation tissue and scaled for the removal of any residual calculus.

Pocket depths were re-evaluated, and bleeding points were marked to identify the base of the periodontal pockets (Figure 1). Internal bevel incisions were placed using a No. 15 blade close to the gingival margin following the contour of the gingiva to excise the pocket epithelium while preserving the maximum amount of healthy gingival tissue. The exact distance from the gingival margin was determined intraoperatively according to the gingival thickness and pocket morphology and extended from the maxillary right central incisor to the first molar (Figure 2). After making crevicular incisions using No. 12, a full-thickness mucoperiosteal flap was carefully reflected only to the extent required to preserve as much soft tissue as possible while allowing sufficient visualization of the root surfaces and alveolar crest (Figures 3, 4). Following flap reflection, the alveolar crest was carefully evaluated for osseous morphology. Localized irregular marginal bony architecture with ledges and non-physiologic contours was identified, which could compromise flap adaptation and maintenance. Conservative osseous recontouring was therefore performed to re-establish a physiologic bony architecture and facilitate optimal soft tissue adaptation. Also, inflamed pocket epithelium and granulation tissue were thoroughly removed (Figure 5). Root surfaces were instrumented using periodontal curettes to eliminate residual calculus and contaminated cementum (Figure 6).

**Figure 7:**
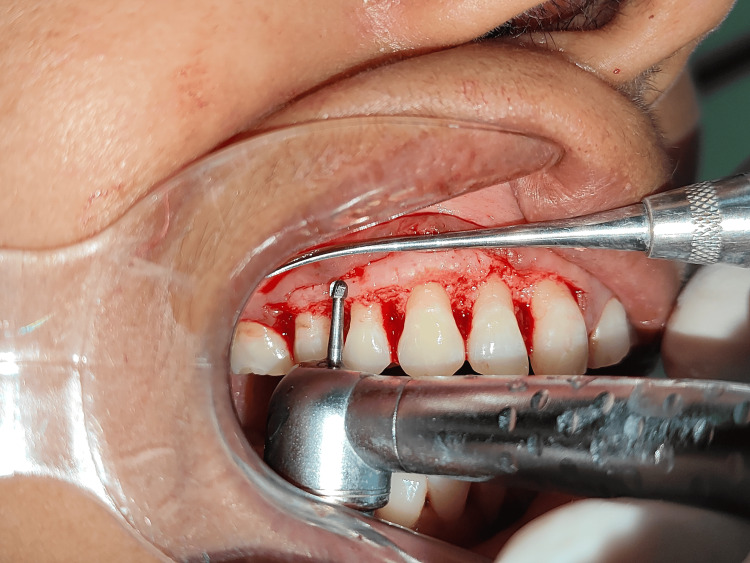
Osseous contouring was done using a round carbide bur with copious irrigation to restore physiologic bony contours and better flap adaptation.

**Figure 8:**
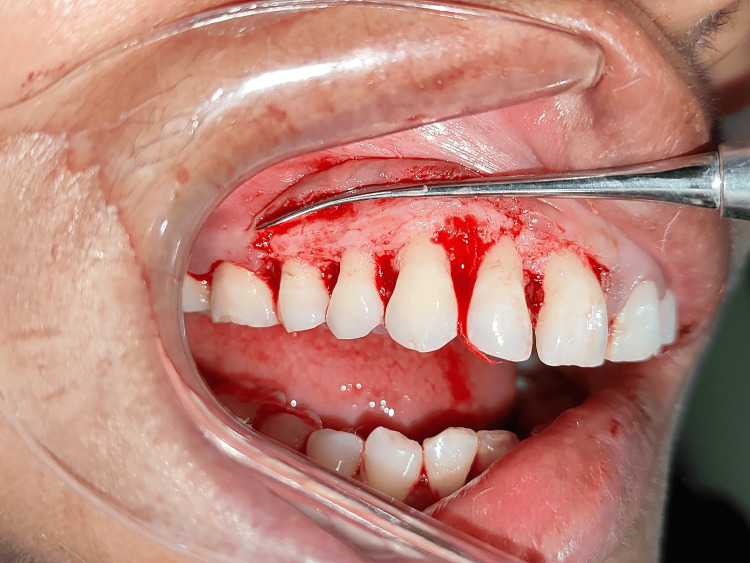
Physiologic bony architecture was restored after osseous recontouring.

**Figure 9:**
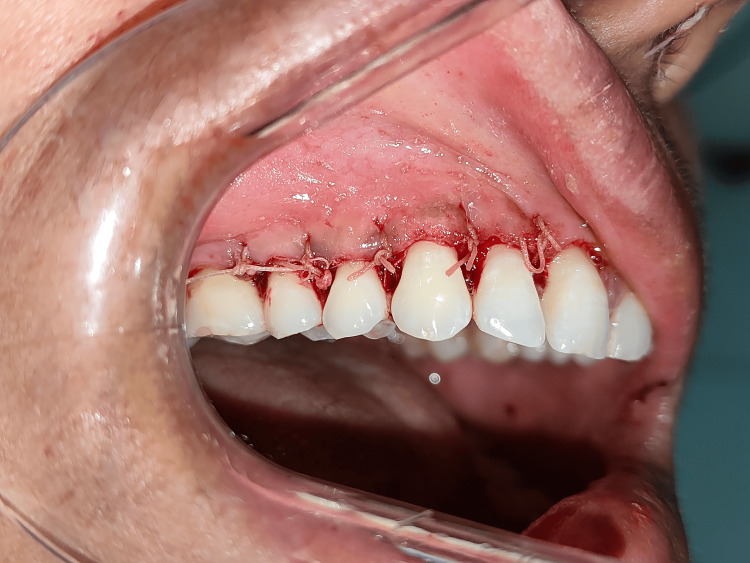
The flap was then repositioned to achieve close adaptation to the root surface and stabilized using interrupted 3-0 polyglycolic acid/polylactic acid (PGA/PLA) sutures without intentional apical displacement.

Following flap reflection, the alveolar bone was carefully evaluated for osseous morphology. Localized irregular marginal osseous architecture compromising physiologic bony contour was observed. Conservative osseous recontouring was therefore performed to re-establish a physiologic osseous architecture and facilitate optimal soft tissue adaptation (Figures 7, 8). The flap was repositioned to its original position and secured using 3-0 polyglycolic acid/polylactic acid (PGA/PLA) sutures. Hemostasis was achieved, and postoperative instructions were provided (Figure 9).

The patient was prescribed systemic amoxicillin-clavulanic acid (625 mg) postoperatively as an adjunct to the surgical procedure to minimize the risk of postoperative infection during the early healing phase and to promote uneventful wound healing; a combination analgesic containing paracetamol (325 mg), diclofenac (100 mg), and serratiopeptidase (15 mg); and, as part of the standard postoperative protocol, the patient was advised to use 0.2% chlorhexidine mouthwash. However, due to poor compliance during the immediate postoperative period (burning sensation), chlorhexidine was temporarily substituted with povidone-iodine mouthwash to facilitate chemical plaque control. Following suture removal, the patient was re-motivated regarding postoperative oral hygiene, became compliant with 0.2% chlorhexidine mouthwash, and continued its use for the remainder of the healing period [10].

**Figure 10:**
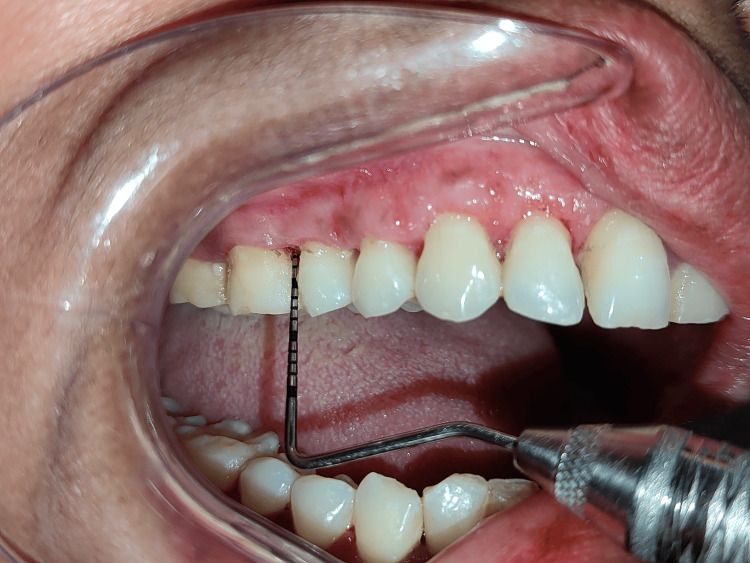
At three months postoperatively, pocket probing depth was measured using a University of North Carolina-15 (UNC-15) periodontal probe, revealing a pocket depth of 3 mm.

A reduction of 2 mm in probing pocket depth and a clinical attachment gain of 2 mm were observed. Minimal postoperative gingival recession was detected. The patient remained asymptomatic and showed improved periodontal health. The patient was also assessed for postoperative dentinal hypersensitivity during follow-up visits, and no clinically significant hypersensitivity was reported (Figure 10, Table 1).

**Table 1:** Clinical parameters pre- and post treatment

Parameter	Baseline	Three Months
Probing Pocket Depth	5 mm	3 mm
Clinical Attachment Level	4 mm	2 mm
Bleeding on Probing	Present	Absent
Gingival Recession	Absent	Minimal/Clinically Insignificant

## Discussion

Residual periodontal pockets following non-surgical periodontal therapy remain a significant clinical challenge because they contain pathogenic microorganisms and increase the risk of disease recurrence [3]. Surgical intervention is usually indicated when adequate root debridement cannot be achieved through non-surgical means alone [4].

The modified Widman flap procedure was selected in the present case because it provides excellent access for instrumentation while minimizing unnecessary soft tissue removal. Ramfjord and Nissle originally described the technique as a conservative surgical approach designed to preserve as much healthy soft tissue as possible while allowing excision of the pocket epithelium and inflamed connective tissue, thereby minimizing unnecessary tissue removal compared with more extensive resective procedures [5].

In the present case, probing pocket depth was reduced from 5 mm at baseline to 3 mm after three months, accompanied by a clinical attachment gain of 2 mm. These findings are consistent with previous investigations demonstrating significant pocket reduction and attachment gain following modified Widman flap surgery [6, 7].

The reduction in probing depth observed in the present case may be due to the elimination of inflamed pocket epithelium, reduction in diseased tissue, and close adaptation of the flap to the root surface following proper debridement [11]. Histological studies have demonstrated that healing following modified Widman flap surgery primarily occurs through the formation of a long junctional epithelium with maturation of the connective tissue attachment over several weeks [12]. Although the present report demonstrated favorable early clinical outcomes at three months, longer follow-up periods are required to evaluate the long-term stability of periodontal attachment and pocket reduction.

Osseous recontouring was performed to establish physiologic bone contours and eliminate any irregular bony contours. According to Ochsenbein, physiologic osseous architecture is essential for maintaining periodontal health and preventing recurrence of periodontal pockets [9]. Osseous recontouring contributes to favorable flap adaptation and facilitates effective plaque control during maintenance therapy [13].

An important observation in the present case was the presence of minimal clinically nonsignificant postoperative gingival recession despite flap reflection and osseous surgery. Gingival recession is a recognized consequence of periodontal surgery and may compromise esthetics, particularly in the anterior region [14]. Careful flap handling, preservation of tissue thickness, and precise flap adaptation may have contributed to the maintenance of gingival architecture observed in this patient. Surgical management of persistent periodontal pockets should be done according to defect morphology, pocket depth, and treatment objectives. Conventional surgical options include open flap debridement, modified Widman flap, apically positioned flap with or without osseous resective surgery, and regenerative procedures. Open flap debridement primarily provides access for root debridement with minimal alteration of the gingival architecture, whereas apically positioned flap surgery is indicated when pocket elimination is prioritized but may result in greater gingival recession and compromised esthetics. Conversely, regenerative procedures are reserved for defects with favorable morphology, such as contained intrabony defects, where regeneration of the periodontal attachment apparatus is biologically feasible. In the present case, no contained intrabony defect suitable for regenerative therapy was identified. Therefore, a modified Widman flap combined with conservative osseous recontouring was considered the most appropriate treatment approach because it provided adequate surgical access for meticulous root debridement, allowed correction of localized osseous irregularities, and facilitated close flap adaptation while preserving as much healthy soft tissue as possible.

Bleeding on probing was absent at the three-month follow-up examination, indicating resolution of periodontal inflammation and achievement of periodontal stability. The clinical outcomes obtained in this report support the success of the modified Widman flap surgery as a predictable and effective treatment modality for residual periodontal pockets following Phase I periodontal therapy. Although the findings were positive, they are based on a single case with a short follow-up period. Long-term clinical studies with larger sample sizes are required to further accept these observations [15].

## Conclusions

In the present case, modified Widman flap surgery combined with conservative osseous recontouring resulted in favorable early clinical outcomes, including reduction in probing pocket depth, gain in clinical attachment, and resolution of bleeding on probing at the three-month follow-up. The case highlights the importance of appropriate case selection and surgical decision-making in the management of persistent periodontal pockets following non-surgical periodontal therapy. However, as this report represents a single clinical case with a limited follow-up period, further studies with larger sample sizes and longer observation periods are required before broader clinical conclusions can be drawn.
